# Interleukin-1β has trophic effects in microglia and its release is mediated by P2X7R pore

**DOI:** 10.1186/s12974-016-0621-8

**Published:** 2016-06-30

**Authors:** Mastura Monif, Christopher A. Reid, Kim L. Powell, Katherine J. Drummond, Terrence J. O’Brien, David A. Williams

**Affiliations:** Department of Physiology, Faculty of Medicine, Dentistry and Health Sciences, The University of Melbourne, Melbourne, Victoria 3010 Australia; Howard Florey Institute, The University of Melbourne, Melbourne, Victoria 3010 Australia; Department of Medicine, Royal Melbourne Hospital, The University of Melbourne, Melbourne, Victoria 3010 Australia; Department of Surgery, Royal Melbourne Hospital, The University of Melbourne, Melbourne, Victoria 3010 Australia; The Department of Neurology, The Royal Melbourne Hospital, Parkville, Victoria 3052 Australia

**Keywords:** Microglia, P2X7 receptor, Interleukin-1β, Activation, Proliferation, P2X7R pore, Neuroinflammation

## Abstract

**Background:**

Enhanced expression of the purinergic P2X7 receptor (P2X7R) occurs in several neuroinflammatory conditions where increased microglial activation is a co-existing feature. P2X7 receptors can function either as a cation channel or, upon continued stimulation, a large pore. P2X7R-over-expression alone is sufficient to drive microglial activation and proliferation in a process that is P2X7R pore dependent, although the biological signaling pathway through which this occurs remains unclear. Once activated, microglia are known to release a number of bioactive substances that include the proinflammatory cytokine interleukin-1β (IL-1β). Previous studies have linked P2X7R stimulation to the processing and release of IL-1β, but whether the channel or pore state of P2X7R is predominant in driving IL-1β release is unknown and is a major aim of this study. In addition, we will determine whether IL-1β has trophic effects on surrounding microglia.

**Methods:**

Electron microscopy and immunohistochemistry were used to delineate the sub-cellular localization of P2X7R and IL-1β in primary hippocampal rat cultures. FM1-43 fluorescent dye and confocal microscopy were used to quantify vesicular exocytosis from microglia expressing the pore-forming P2X7R versus a non-pore-forming point mutant, P2X7R*G345Y*. IL-1β in culture was quantified with an enzyme-linked immunosorbent assay (ELISA). IL-1β intracellular processing was blocked with inhibition of caspase 1 (with a synthetic peptide antagonist), and its extracellular form was neutralized with an IL-1β neutralizing antibody. Microglial activation and proliferation was quantified immunohistochemically with confocal microscopy.

**Results:**

P2X7R and IL-1β were co-localized in lysosomes. Vesicular exocytosis was higher in microglia expressing the pore-forming P2X7R compared to those expressing the non-pore-forming mutant. There was increased IL-1β in cultures expressing the pore-forming P2X7R, and this proinflammatory cytokine was found to mediate the trophic effects of P2X7R pore in microglia. Inhibition of IL-1β production and function resulted in a significant decrease in P2X7R-mediated microglial activation and proliferation.

**Conclusions:**

IL-1β is a mediator of microglial activation and proliferation, and its release/production is P2X7R pore dependent. Blockade of P2X7R pore could serve as a therapeutic target in alleviating the degree of inflammation seen in neurodegenerative and neoplastic conditions.

**Electronic supplementary material:**

The online version of this article (doi:10.1186/s12974-016-0621-8) contains supplementary material, which is available to authorized users.

## Background

The pathogenesis of a number of diseases such as Alzheimer’s, Parkinson’s, multiple sclerosis, and human gliomas are thought to be a result of chronic neuroinflammatory processes. Microglial cells that exhibit specialized immune characteristics in the central nervous system play a crucial role in neuroinflammation. In a healthy CNS environment, microglia exhibit a quiescent morphology. However, under pathological conditions and in the setting on neuroinflammation, they undergo a process known as microgliosis. In this situation, microglia become activated with an enlarged soma, and acquisition of lamellapodia. They up-regulate the synthesis of a number of bioactive substances such as cytokines. The identity, amount, and temporal expression profile of these cytokines determines if the neuroinflammatory process are neuroprotective or ultimately lead to neurodegeneration [1].

Interleukin-1β (IL-1β) is a proinflammatory cytokine released by activated macrophages and microglia. There are numerous reports highlighting that the release of IL-1β from immunological cells is dependent on the activity of P2X7 receptor (P2X7R) [2–12], a purinergic receptor that is expressed in cells of hemopoeitic and immunological origin (including monocytes, macrophages, and microglia). The stimulation of P2X7R allows a significant efflux of K^+^, which favors the formation of the active form of caspase 1 [13], leading to production of mature IL-1β. P2X7R can therefore modulate inflammation by influencing cytokine production.

An enigmatic feature of P2X7R is its dual conductance states. Brief stimulation of P2X7R with agonists such as ATP or 2′3′-*O*-(4-benzoylbenzoyl)-ATP (BzATP) leads to opening of the P2X7R channel permeable to small cations. However, sustained stimulation leads to formation of a P2X7R pore permeable to moieties of up to 900 Da. The physiological basis and biological role of P2X7R pore formation is currently unresolved. Interestingly, polymorphisms in P2X7R are reported to alter cytokine production. One such polymorphism, Glu496Ala, leads to loss of function of P2X7R [14], with reduced ATP-stimulated K^+^ efflux and delayed release of IL-1β [9]. While P2X7R is clearly important in the production/release of IL-1β, the relative contributions of channel or pore conductance and the exact mode of release of the cytokine are unknown. IL-1β does not contain signal sequence for compartmentalization within the Golgi or the classical secretory pathway; therefore, pro-IL-1β accumulates within the cytosol [15]. IL-1β is thought by some to be released via simple budding of the plasma membrane into small membrane-delimited microvesicles [16], while others have reported release via small vesicles named exosomes (derived from exocytosis of multivesicular bodies) [17] or to be simply subsequent to cell death [15]. Release via lysosomal vesicular exocytosis has also been suggested [18].

P2X7R over-expression is sufficient to drive microglial activation and proliferation, trophic responses that were shown to be dependent on the pore activity of P2X7R [19, 20]. Similarly, in human glioma cultures, P2X7R over-expression in glioma-associated microglia and subsequent inhibition of P2X7R were sufficient to reduce glioma cell number [21]. There is an established link between P2X7R function and the release/production of the proinflammatory mediator IL-1β [2, 4, 5, 16]. Here, we reveal that IL-1β release is P2X7R pore dependent and that the blockade of the P2X7R pore could serve as a therapeutic option for alleviation of inflammation.

## Methods

### DNA constructs and site-directed mutagenesis.

Two enhanced green fluorescent protein (EGFP) fusion constructs used in this study for transfection of primary hippocampal astrocyte-microglia mixed cultures were as follows: P2X7R-EGFP where the wild type rat P2X7R (accession number: X95882) was fused to the N terminus of EGFP and P2X7R*G345Y*-EGFP where a point mutant of P2X7R was fused to the N terminus of EGFP as previously described [20]. This mutant, P2X7R*G345Y*-EGFP, was produced by targeting the glycine at position 345 in the rat P2X7R for substitution with tyrosine using a QuickChange Site-Directed Mutagenesis Kit (Stratagene) with the following primers:

Forward: 5′C ACC CTG TCC TAT TTC **TA**T TTG GCC ACC GTG TG

Reverse: 5′ CA CAC GGT GGC CAA A**TA** GAA ATA GGA CAG GGT G

Base changes introducing the *G*345 to *Y* mutation are in bold type and underlined. All constructs were expressed under the influence of CMV promoter.

### Primary hippocampal neuron-glia mixed cultures

Protocols for handling animals were reviewed and approved by the Animal Ethics Committee at The University of Melbourne, Australia. Primary hippocampal neuron-glia mixed cultures were prepared from P2-5 Sprague-Dawley rats as described previously [22]. Briefly, the animals were anesthetized by halothane inhalation, the brains were removed, and the hippocampi were dissected out and finely chopped. The hippocampal pieces were placed in an enzyme solution containing papain (200 units; Sigma-Aldrich) for 35 min at 37 °C. The hippocampal tissue was washed three times to remove all traces of papain, and the mixture was triturated to obtain a single cell suspension. The cells were plated into 12-well plates containing 18-mm poly-d-lysine (Sigma) coated coverslips (SDR Clinical Technology) at a density of 1.8 × 10^5^ cells/well. The cultures were maintained in Minimum Essential Medium (Gibco, Invitrogen) with the following supplements: 1 mM glucose, penicillin-streptomycin (5000 units/mL), 10 % heat inactivated fetal bovine serum (Gibco, Invitrogen), MITO+™ Serum Extender (Becton Dickinson), and 2 mM L-glutamine (Gibco, Invitrogen). The cells were cultured at 37 °C in a humidified incubator of 5 % CO_2_/95 % O_2_. Untransfected cultures contained ~48 % astrocytes and ~50 % microglia as assessed by immunohistochemistry using antibodies against glial fibrillary acidic protein (GFAP) and isolectin GS-IB_4_, respectively.

### Microglia-enriched cultures

Initially, neuron-glia mixed cultures were prepared in 75 cm^2^ flasks (JRH Biosciences). One animal was used per 75 cm^2^ flask. After 14 days, the flasks of mixed neuron-glia cultures were shaken (Economy Orbital Mixer, U-lab) at 150 rpm for 4 h at 37 °C, to dislodge microglia loosely attached to underlying astrocytes. The medium containing microglia was then aspirated and centrifuged at 1000 rpm for 5 min. The pellet of microglia was re-suspended in supplemented culture medium and placed in 12-well plates containing poly-d-lysine-coated coverslips. One 75 cm^2^ flask of mixed cultures was used for preparing four coverlips (wells) of a 12-well culture plate. There were 3.1 × 10^4^ microglia/coverslip at 24 h post-harvest. The media was changed once a week, and the cells were maintained in the incubator for further 1 week before use in the experiments. Purity was assessed by labeling with the microglial maker, isolectin GS-IB_4_, which identified 94 % of cells as microglia. Enzyme-linked immunosorbent assay (ELISA) experiments were conducted using microglia-enriched cultures to quantify the amount of IL-1β in culture.

### Transfection

The exogenous plasmid DNA constructs, P2X7R-EGFP or P2X7R*G345Y*-EGFP, were individually introduced into cultured cells using a modified calcium phosphate transfection technique [23], with the aid of a CalPhos™ Mammalian Transfection Kit (Becton Dickinson Biosciences) according to the manufacturer’s protocols. The DNA concentration used was 2 μg/well of a 12-well culture plate (1.8 × 10^5^ cells/well). The expression of the exogenous constructs was monitored with an Olympus IX 81 fluorescence microscope 72 h post-transfection. This time was sufficient for the folding and trafficking, which are identical for each receptor type, and for the divergence of downstream effects of each exogenous P2X7R. The transfection efficiency was 20 %.

### Immunohistochemistry

At 72 h post-transfection, neuron-glia mixed cultures were fixed for 15 min in a 4 % paraformaldehyde solution at room temperature. After fixation, the cells were washed once with phosphate-buffered saline (PBS), and non-specific protein binding sites were blocked with 2 % bovine serum albumin (Sigma) for 45 min at 37 °C. The following primary antibodies were used: mouse anti-GFP (final dilution, 1:200; Molecular Probes), anti-LAMP1 primary antibody (final dilution of 1:200; Abcam), isolectin GS-IB_4_ from *Griffonia simplicifolia*, Alexa Fluor® 594 conjugate (final dilution of 1:100; Molecular Probes), anti-IL-1β (MAB501; final dilution, 1:100; Abcam), and OX-6 (anti-MHC II) (final dilution, 1:100; BD Pharmingen). Primary antibodies were made up in PBS, with 1 % Triton X-100 for permeabilization, and were incubated overnight at 4 °C. After three 5-min washes in PBS, the sections were incubated with the relevant secondary antibodies: Alexa Fluor® 488 (final dilution, 1:200; Molecular Probes) or Texas Red® X (final dilution, 1:200; Molecular Probes). All secondary antibodies were incubated overnight at 4 °C. After three 5-min washes in PBS, the samples were mounted with DAKO Fluorescent Mounting Medium. No staining was detected in the absence of the primary or secondary antibodies. Some preparations were counter-labeled with DAPI nuclear stain (5 μM; Molecular Probes).

### Confocal microscopy

Seventy-two hours post-transfection, primary mixed cultures were viewed with an Olympus IX-81 fluorescence microscope equipped with a DG4 light source. Images were acquired with a 40× LucPlanFI Olympus (N.A. 0.6) air objective. For most experiments, the samples were simultaneously stained with two or three fluorescent probes, with dual or triple emission achieved through appropriately selected emission filters. Images were analyzed using MetaMorph (Universal Imaging Corporation®) software for assessment of microglial activation and proliferation. For live cell imaging, the cells were bathed in HEPES buffer (mM: NaCl 135, KCl 5, HEPES 10, Glucose 10, CaCl_2_ 1, MgCl_2_ pH 7.4) at room temperature (~25 °C).

### Electron microscopy

The primary hippocampal neuron-glia mixed cultures were seeded onto thermanox coverslips (ProScitech). Seven days post culture, the cells were transfected with various DNA constructs. The transfected coverslips were fixed with 4 % paraformaldehyde and 0.1 % glutaraldehyde for 10 min at room temperature and transferred to the EM facility (The University of Melbourne) for embedding and grid sectioning (thin sections). Thin cryosections were prepared from glutaraldehyde and/or paraformaldehyde-fixed tissue after infusion with 2.3 M sucrose. The sections were then composed on carbon-coated nickel and picked up on a solid layer of 2 % gelatin in buffer. The grids were then prepared for immunohistochemistry.

On a sheet of wax (paraffin) for each grid, a ~0.5-mL drop of blocking buffer (1 % BSA made in PBS) was placed. With fine forceps, the grids were individually positioned on top of the buffer (cells facing up) floating in the droplet. Non-specific binding sites were blocked for 10 min at room temperature. The grids were then transferred to drops of primary antibody (1:50 final dilution, anti-GFP antibody; Molecular Probes). The primary antibody was made up of PBS with 0.1 % Tween 20 (Amresco). The grids were incubated with primary antibody for 24 h at 4 °C, washed (three times) by gently transferring them to drops of washing buffer (sodium cacodylate trihydrate; Sigma), and then incubated for 2 h with drops of secondary antibody, anti-mouse IgG (whole molecule) gold conjugate, 10 nm (Sigma), diluted just before use to a concentration of 1:50. The grids were placed in a washing buffer (2 × 10 min washes), and the cells post fixed in 2 % glutaraldehyde in PBS and then washed with cacodylate buffer, for 3 × 2 min.

The grids were returned to the EM facility, stained with uranyl acetate in veronal buffer (pH 6.4) for 15 min, washed in water for 3 × 2 min, and dehydrated through 40 %, 75 %, 95 %, and 2 × 100 % ethanol for 2 min each. They were then left in 50/50 ethanol/LR (London resin) white for 10 min, then left in 100 % LR white for 2 × 10 min, and then sandwiched between Whatman filter papers with light pressure applied to remove residual liquid. After 1–2 h in an oven at 60 °C grids were counterstained with conventional uranyl acetate and lead citrate. The cells were viewed with an electron microscope with a minimum ×2000 magnification. Negative controls without primary antibody were generated for all experiments, and no signal was obtained.

### Assessment of microglial activation

Microglial activation was assessed using morphological and immunohistochemical criteria as previously described [20]. Before analysis of activation, microglia were classified into two groups. Cells with an oval cell body containing a small volume of cytoplasm and long, thin, delicate, and radially branched processes were classified as ramified microglia [24]. Activated microglia were defined as having an enlarged soma (width greater or equal to 30 μm) [25, 26] and a broad-flattened appearance with the common presence of several lamellapodia [27]. This morphological classification was confirmed immunohistochemically by the use of microglial specific markers isolectin GS-IB_4_ and CD-11b.

### Assessment of microglial proliferation

We used isolectin GS-IB_4_ as previously described [28], to quantify microglial proliferation. The transfected cells were identified with anti-GFP and a secondary Alexa Fluor® 488 antibodies. Microglia were selectively identified with isolectin GS-IB_4_ staining, and all nuclei were stained with DAPI (Molecular Probes). Images were analyzed using MetaMorph (Universal Imaging Corporation®) software for assessment of proliferation.

### Assessment of microglial exocytosis- FM1-43 experiments

Shedding of vesicular contents (exocytosis) from microglia expressing the pore- or non-pore-forming P2X7R was visualized by labeling the cells with the membrane permeant dye FM1-43 and measuring the changes in cell surface fluorescence upon P2X7R stimulation. ATP and BzATP were used to stimulate P2X7R. As well as labeling of vesicles and vesicular contents, the uptake of the styryl (FM) dyes is generally utilized in the study of dynamics of vesicular release (endo- and exocytosis) [18]. FM1-43 is known to stain lysosomes and reveal lysosomal vesicular recycling [29]. Upon vesicular release, the cell surface FM1-43 fluorescence is expected to increase as the intracellular vesicle that is docking just inside the cell fuses with the cell membrane [30]. Thereafter, the membrane fluorescence is expected to transiently decrease as the contents are released.

The *x,y* coordinates of activated microglia expressing P2X7R-EGFP or P2X7R*G345Y*-GFP were recorded for later imaging and all cells were loaded with 5 μM of FM1-43 for 5 min while the cells were bathed in PBS. Then, the cells were washed with BPS and incubated in HEPES buffer.

Fusion events were monitored as a time series of cell and the plasma membrane fluorescence. The temporal distribution of exocytosis events was obtained by defining regions of interest (ROI) of the cell surface and monitoring changes in membrane fluorescence using “TimeSeries” (LSM510 META software). All fluorescence values were corrected for background fluorescence levels.

### Media exchange experiments

At 72 h post-transfection, the supernatant derived from cultures expressing P2X7R-EGFP or P2X7R*G345Y*-EGFP was transferred to untransfected 7-day-old primary hippocampal cultures, and the cultures were subsequently kept at 37 °C in a humidified incubator of 5 % CO_2_/95 % O_2_ for a further 72 h. Thereafter, the cells were fixed in 4 % paraformaldehyde (10 min) and processed for immunohistochemistry with isolectin GS-IB_4_ to determine the number of microglia and DAPI nuclear stain to quantify the total number of cells.

### IL-1β enzyme linked immunosorbent assay (ELISA)

IL-1β produced in the hippocampal microglia-enriched cultures expressing P2X7R-EGFP or P2X7R*G345Y*-EGFP was quantified with the aid of a Quantikine IL-1β ELISA kit (R&D Systems), according to the manufacturer’s protocols. Briefly, 72 h post-transfection, the cultures expressing P2X7R-EGFP or P2X7R*G345Y*-EGFP were exposed to BzATP (100 μM), a specific agonist of P2X7R, for 30 min. At this time point, detectable amounts of ATP-induced IL-1β release have been previously reported [7]. Supernatants were collected and centrifuged at 2000 rpm for 3 min to pellet cellular debris. Triton X-100 (1 %) was added to the purified supernatants and gently mixed to dislodge IL-1β from the shed microvesicles [16] for assay by ELISA.

### Caspase 1 inhibition experiments

Primary hippocampal cultures expressing P2X7R-EGFP or P2X7R*G345Y*-EGFP at 24 h post-transfection were exposed to N-Acetyl-TRP-GLU-His-Asp-AL (0.67 mg/mL; Sigma-Aldrich), a potent antagonist of ICE (caspase 1) [31, 32]. The inhibitor was left in culture from 24 to 72 h. The 24-h time point allows sufficient time for expression of the exogenous constructs (unpublished findings). At 72 h post-transfection (allowing sufficient time for downstream signaling cascades to develop), the cultures were fixed and processed for immunohistochemistry to measure the degree microglial activation and proliferation.

### IL-1β neutralization experiments

The activity of the mature (extracellular) IL-1β was blocked with a specific IL-1β neutralizing antibody (R&D Systems). At 24 h post-transfection, the cultures expressing P2X7R-EGFP or P2X7R*G345Y*-EGFP were treated with a neutralizing antibody (1:50), and at 72 h post-transfection, the cultures were processed for immunohistochemistry and imaging to quantify microglial activation and proliferation.

## Results

### P2X7R-activated microglia exhibited “nodular structures” containing intraluminal vesicles which expressed P2X7R

**Fig. 1:**
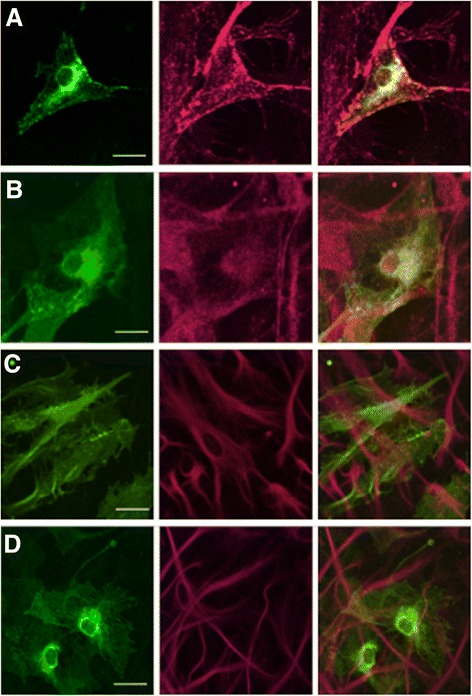
**a** In P2X7R-EGFP-transfected primary hippocampal cultures, the transfected cells co-localized with microglia marker isolectin GS-IB4 (*red*: microglial marker—isolectin GS-IB4, *green*: P2X7-EGFP). **b** Also, in P2X7R-EGFP-transfected primary hippocampal cultures, the transfected cells co-localized with another microglia marker OX-6 anti-MHC II (*red*: microglial marker—OX-6, *green*: P2X7-EGFP). **c, d** No co-localization was observed between cells expressing P2X7R-EGFP and astrocytes (*green*: P2X7R-EGFP, *red*: GFAP—astrocytic marker). *Scale bar*: 5 μm

P2X7R transfection is sufficient to drive microglial activation and proliferation [20]. Primary hippocampal cultures transfected with P2X7-EGFP showed that the cells expressing the exogenous construct co-localized with that of the microglial marker, isolectin GS-IB4 (Fig. 1a) and another maker of activated microglia OX-6 (MHC II) (Fig. 1b). The cells expressing P2X7-EGFP did not co-localize with that of an astrocytic marker, GFAP (Fig. 1b, c).

**Fig. 2:**
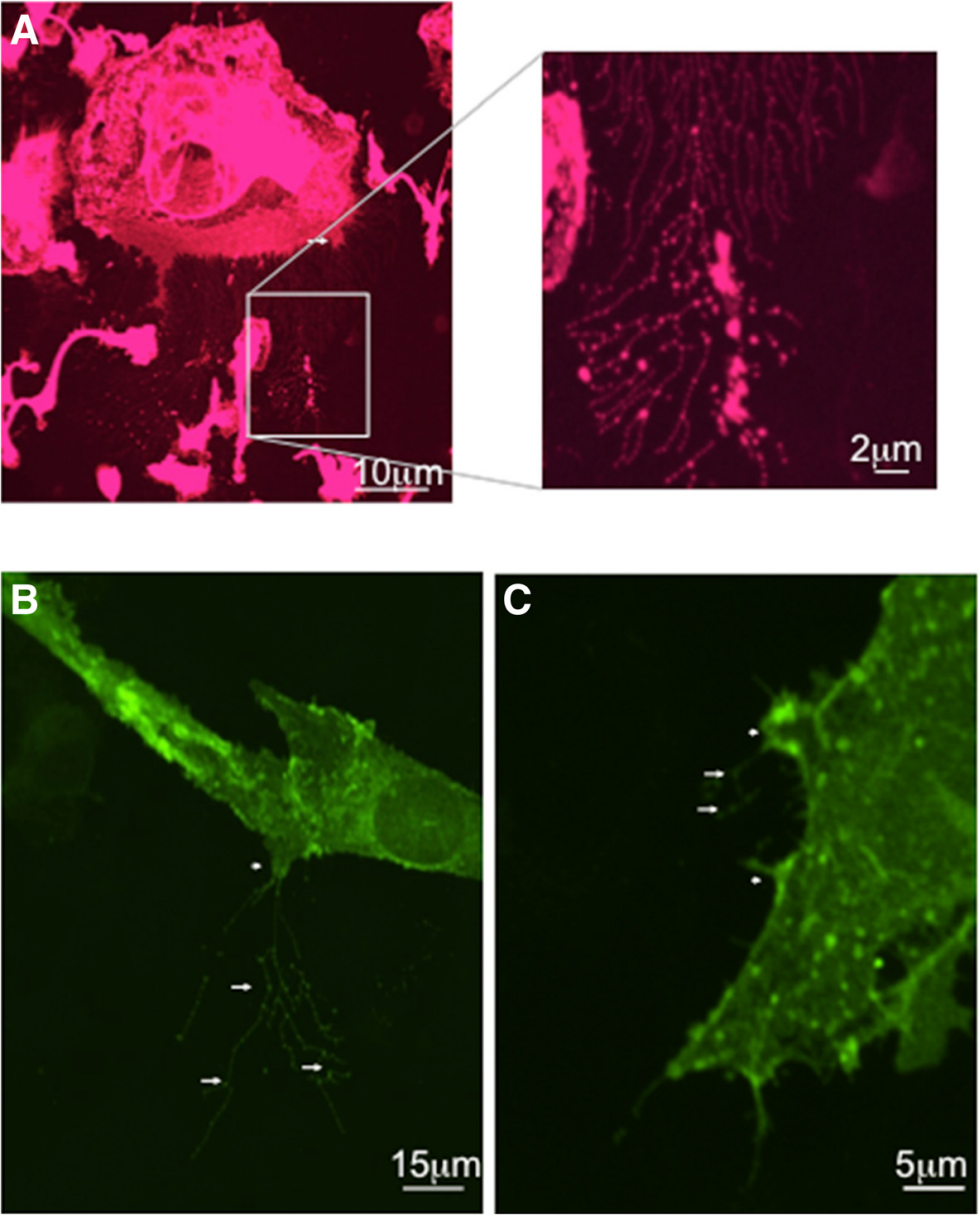
Primary hippocampal cultures expressing exogenous P2X7R exhibited nodular structures as outpouchings of the cell cytoplasm from which emanated a highly organized array of what appeared to be vesicles. **a** Cultures expressing P2X7R stained with microglial marker isolectin GS-IB4 showed nodular structures (*arrow head*) from which projected “beaded” structures to the outside of the cell (*arrows*). *Inset* shows a higher resolution of the “beaded” structures. **b**, **c** Examples of activated microglia expressing exogenous P2X7R-EGFP. As can be noted “nodular” outpouchings of the cell are once again evident with fluorescent “beaded” structures outside the cell. *Scale bar*: as indicated in the figures

“Beaded” structures outside the cell that emanated from “nodular” outpouchings of the cell cytoplasm were evident in microglia (isolectin GS-IB4 positive) expressing exogenous P2X7R (Fig. 2a). Figures 2b, c shows high concentrations of P2X7R expression in the “nodular structures” of activated microglia (Fig. 2b, c).

**Fig. 3:**
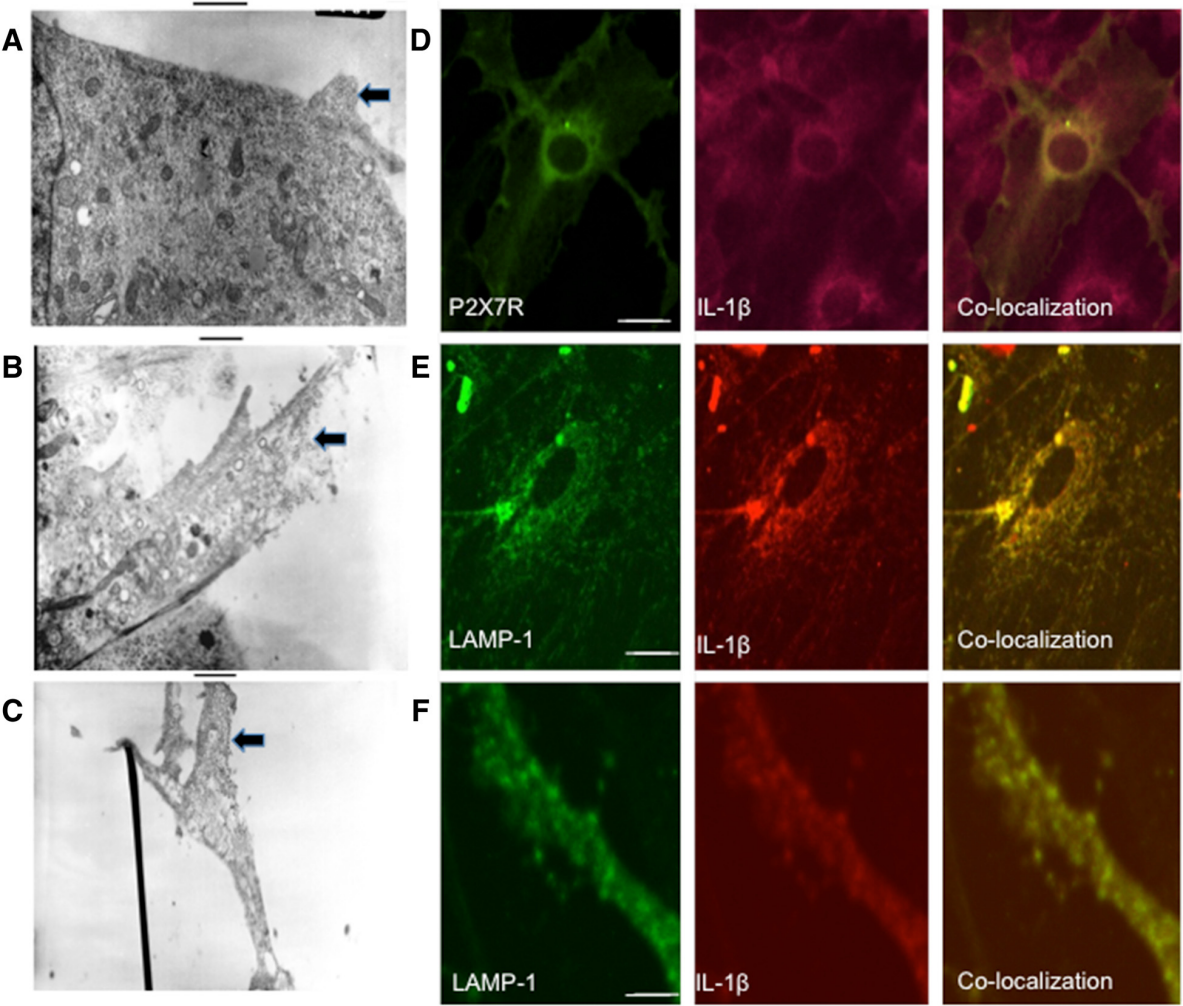
**a**–**c** Electron micrographs of microglia from cultures expressing P2X7R-EGFP. As can be noted within the nodular structures, there resides small vesicles ranging in size from 100 to 200 μm (*arrows*) (**a**) and there is expression of P2X7R within the membrane of these vesicles (**b**, **c**). *Scale bar* 1 μm. **d** The vesicular structures expressing P2X7R were co-localized with expression of IL-1β. *Green*: anti-P2X7R antibody followed by Alexa Fluor 488. *Red*: anti-IL-1β antibody with secondary anti-Texa Red X. *Far right image* shows co-localization of P2X7R and IL-1β. **e**–**f** the vesicular structures expressing P2X7R also expressed LAMP-1 (marker of lysosomal vesicles). *Green*: anti P2X7R antibody followed by Alexa Fluor 488. *Red*: LAMP 1 antibody with secondary anti-Texa Red X. *Far right image* shows co-localization of IL-1β in lysosomes

Electron microscopy revealed that the “nodular structures” contained multiple round intraluminal vesicles ranging in size from 0.1 to 0.2 μm docked near the plasma membrane (Fig. 3a). Immuno-gold revealed that P2X7R receptor expressed on the membrane of vesicles as well as on nearby plasma membrane (Fig. 3b, c).

Immunohistochemical analysis indicates that sub-plasma membrane vesicles expressing P2X7R also expressed IL-1β (Fig. 3d). The expression of IL-1β co-localized with a marker of lysosomes, LAMP-1 (Fig. 3e, f).

### Microglia expressing the pore-forming P2X7R showed a higher degree of vesicular exocytosis

**Fig. 4:**
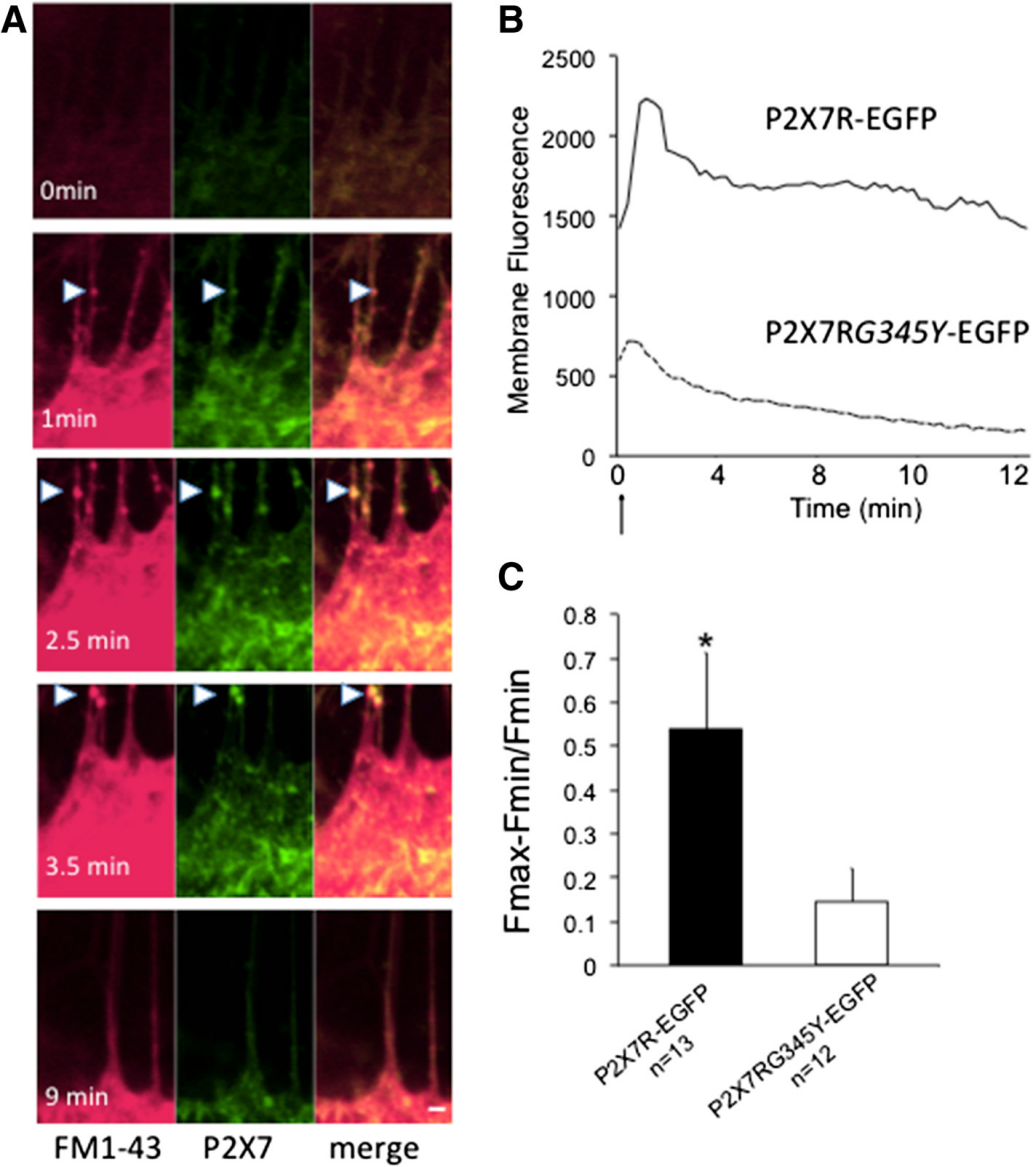
Shedding of vesicular contents (exocytosis) from microglia expressing the pore or non-pore-forming P2X7R was visualized by labeling the cells with the membrane permeant dye FM1-43 and measuring the changes in cell surface fluorescence upon P2X7R stimulation. ATP and BzATP were used to stimulate P2X7R. **a** An activated microglia expressing the pore-forming P2X7R, which was then loaded with FM1-43. *Red*: FM1-43. *Green*: P2X7R-EGF expression. Upon ATP stimulation, there appeared to be the presence of vesicular structures arising on the surface of the cell (*arrows*) which were then released into the extracellular milieu. The images are from 0 to 3.5 min after stimulation with 3 mM of ATP. **b** The cell membrane fluorescence increased with ATP stimulation (coinciding with fusing of the vesicular membrane with cell surface membrane). This was then followed by a decreased in membrane fluorescence. *Arrow* indicates time of application of 3-mA ATP. **c** The change in membrane fluorescence (measured in experiments conducted up to 25 min) in response to ATP (3 mM) was significantly higher in cells expressing P2X7R-EGFP (*n* = 13 cells from 13 different rats) compared to those expressing P2X7R*G345Y*-EGFP (*n* = 12 cells from 12 different rats)

**Fig. 5:**
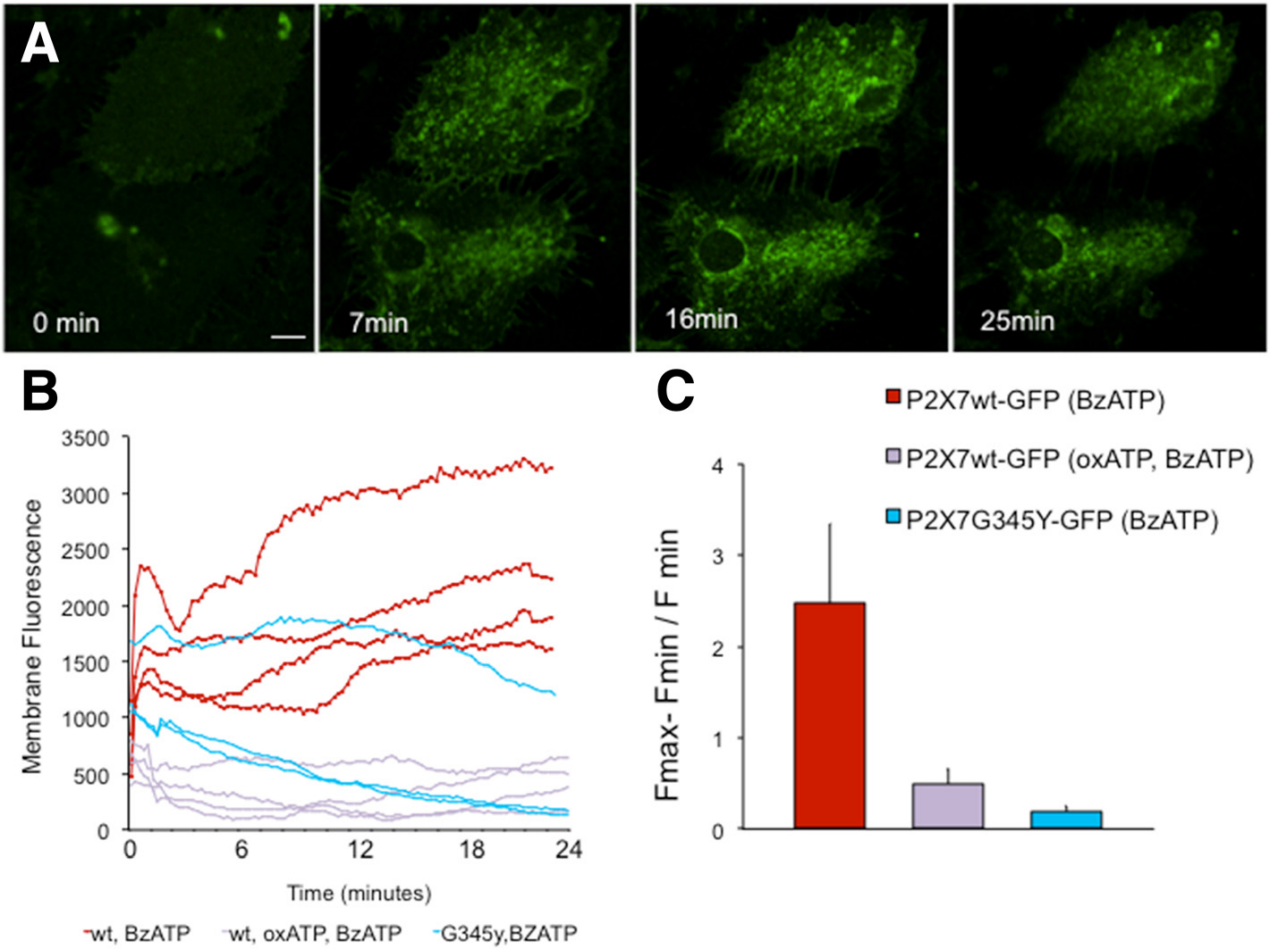
**a** An activated microglia expressing P2X7R-EGFP, subsequently loaded with FM1-43. When this cell was then stimulated with BzATP (100 μM), vesicles of ~200-μm diameter appeared on the surface of the cell which was followed by subsequent “release events” (see Additional file 1 for detailed images of the experiment in real time). The exocytosis was relatively fast to begin with, starting within 15 s of application of the agonist, reaching its maximum within 90–120 s and continuing for a duration of 5 min. **b** Change in membrane fluorescence as a measure of vesicular exocytosis in FM-143-loaded cells in response to 100 μM BzATP. Change in membrane fluorescence from cells expression wild-type P2X7R (P2X7R-EGF; *red line*) and loaded with FM-143 as compared to those expressing the non-pore-forming mutant (P2X7R*G345Y*-EGFP; *blue lines*) as compared to those expressing wild-type P2X7R (P2X7R-EGF) but pre-treated with oxATp (*purple lines*) (100 μM). **c** The sum of exocytosis was presented as change in membrane fluorescence. In response to 100 μM BzATP, there was a significantly higher amount of vesicular exocytosis from microglia expressing the pore-forming wild-type P2X7R (P2X7R-EGF) as compared to those expressing the non-pore-forming mutant (P2X7R*G345Y*-EGFP) or those expressing the wild-type P2X7R with prior inhibition with P2X7R antagonist, oxATP (100 μM)

Exocytosis was measured with FM1-43, a cell permeant fluorescent dye that loads lysosomal vesicles [29]. Figure 4a shows an activated microglia expressing wild-type P2X7R and stained with FM1-43. ATP caused an initial increase in plasma membrane fluorescence (indicating fusion of sub-plasmalemmal vesicles with the cell surface) and subsequent decrease in fluorescence (indicating extracellular release of vesicular contents) (Fig. 4b). The change in membrane fluorescence in response to ATP (3 mM) was significantly higher in cells expressing P2X7R-EGFP compared to those expressing P2X7R*G345Y*-EGFP (Fig. 4c), indicating that the cells expressing the pore-forming P2X7R were undergoing more exocytosis (release events/unit time). To confirm that the response was P2X7R specific, the experiment was repeated using BzATP (specific P2X7R agonist [33]) and oxATP (specific P2X7R antagonist, [34]). Figure 5a shows that when stimulated with BzATP, vesicles of ~200 μm diameter appeared on the surface of the cell followed by subsequent “release events” (see Additional file 1 for detailed images of the experiment in real time). The fused puncta often faded within 100–180 s. In response to BzATP, there was a significantly more vesicular exocytosis from microglia expressing the pore-forming wild-type P2X7R than those expressing the non-pore-forming mutant (*G345Y*) (Fig. 5b, c). This further confirmed that P2X7R pore is involved in exocytosis events in microglia and that the response is P2X7R specific, as it was blocked with oxATP.

### Media derived from primary hippocampal cultures expressing the pore-forming P2X7R contain a factor that is trophic to microglia

**Fig. 6:**
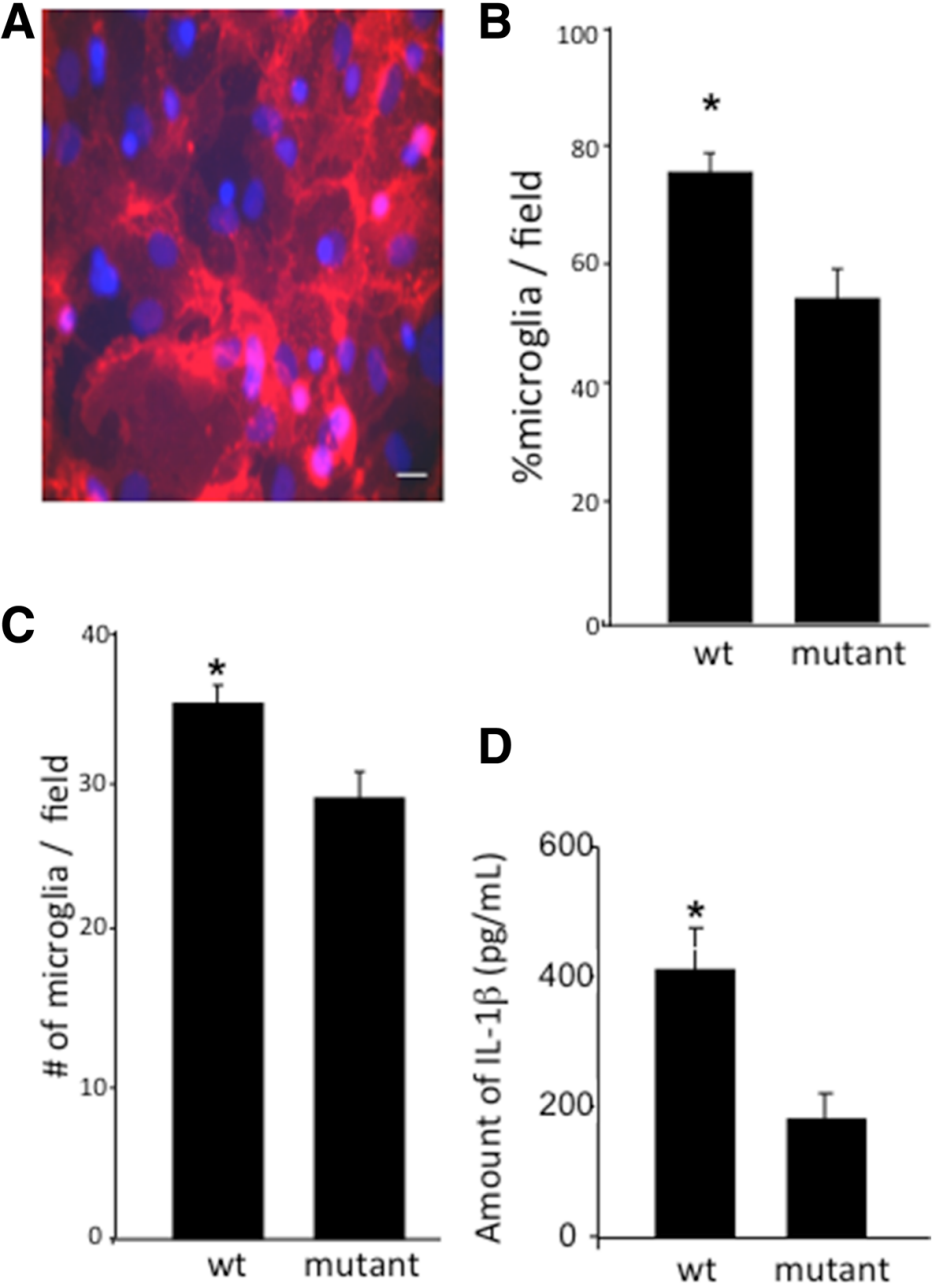
Media derived from primary hippocampal cultures expressing the pore-forming P2X7R contain a factor trophic for microglia. **a** Representative image of untransfected primary hippocampal cultures exposed for 72 h to the supernatant derived from cultures expressing P2X7R-EGFP. *Red*, isolectin GS-IB_4_; *blue*, DAPI nuclear stain. *Scale bar*, 10 μm. **b** The percentage of microglia (isolectin GS-IB_4_-positive cells) over the total number of cells (DAPI-positive cells) per imaging field was increased in cultures exposed to P2X7R-EGFP-derived supernatant compared to P2X7R*G345Y*-EGFP supernatant. **c** The absolute number of microglia (isolectin GS-IB_4_-positive cells) per imaging field was increased in cultures exposed to P2X7R-EGFP-derived supernatant compared with P2X7R*G345Y*-EGFP supernatant. Cell numbers were determined from 49 and 48 randomly selected fields (*x*–*y* dimensions: 230 × 230 μm), respectively, from at least three independent experiments, 72 h post exposure to the supernatants. Data are mean ± SEM. **d** ELISA for IL-1β shows significantly higher levels of the proinflammatory cytokine release in primary hippocampal cultures expressing P2X7R-EGFP (*n* = 6) than in those expressing P2X7R*G345Y*-EGFP (*n* = 6). The absorbance data were measured in duplicates. The results show mean ± SEM. *p < 0.05 (Student’s *t* test)

Media from untransfected hippocampal cultures were replaced with the supernatants from cultures expressing either wild-type or mutant non-pore-forming P2X7R (P2X7R*G345Y*-EGFP). The absolute number and the proportion of microglia in culture were increased by supernatant derived from cultures expressing the wild-type construct compared to the mutant (Fig. 6a–c). This suggested that P2X7R pore-mediated biological “factor(s)” contained in the media were capable of driving microglial proliferation in untransfected cultures.

In response to BzATP (100 μM) stimulation, the extracellular medium from cultures expressing the wild-type P2X7R-EGFP contained a significantly higher amount of IL-1β, than that from cultures expressing non-pore-forming P2X7R*G345Y*-EGFP (Fig. 6d). If IL-1β is a potential facilitator of the P2X7R-mediated trophic effects on microglia, we would expect that inhibiting the action, or reducing the levels, of IL-1β would reduce the level of P2X7R-mediated microglial activation and proliferation of microglia.

### Microglial activation is inhibited following the blockade of IL-1β processing to its active form

**Fig. 7:**
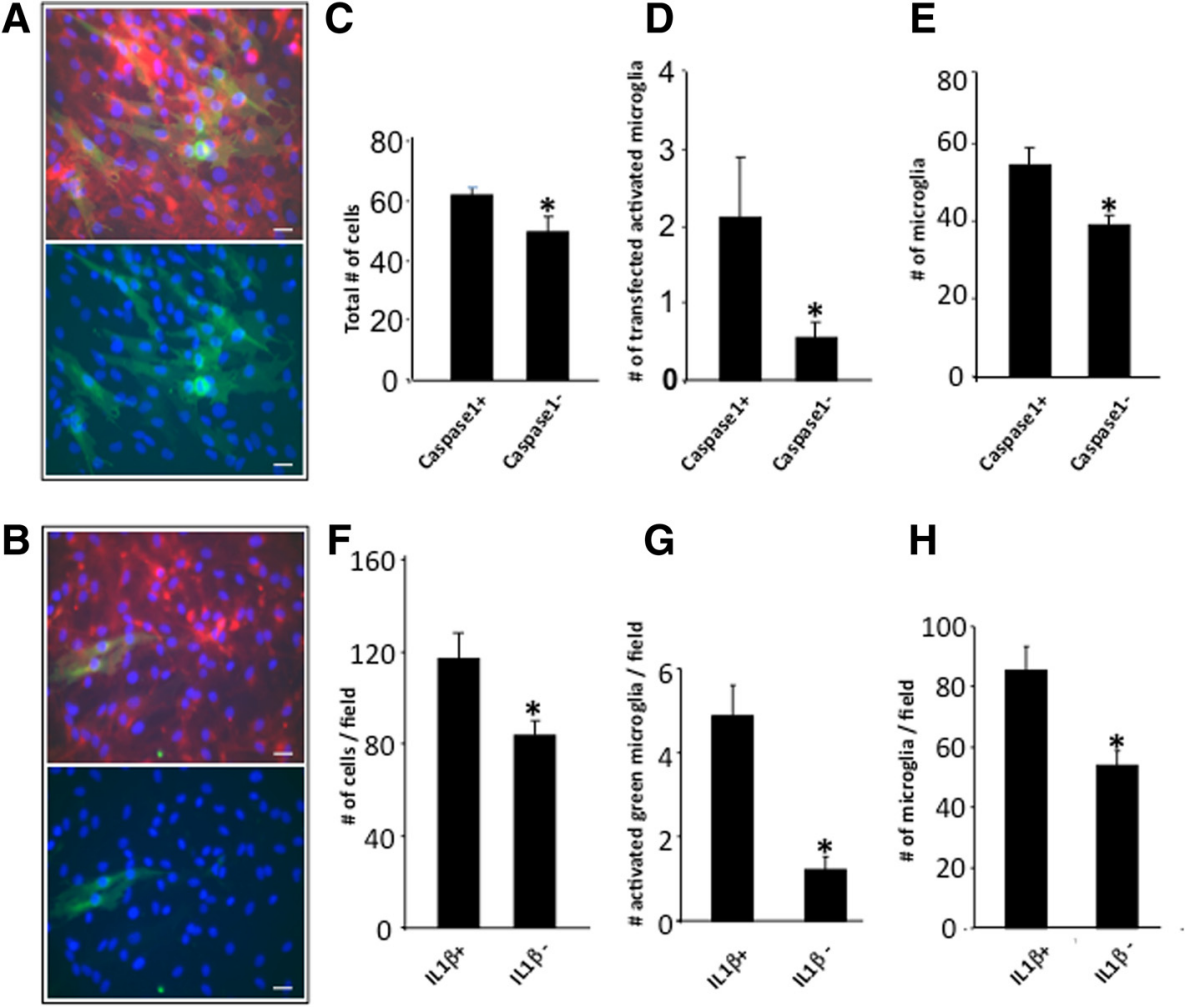
Inhibition of interleukin converting enzyme caspase 1 (with N-Acetyl-TRP-GLU-His-Asp-AL at 0.67 mg/mL) or neutralization of IL-1β activity decreases microglial activation. Representative images from cultures expressing P2X7R-EGFP without (**a**) or with (**b**) IL-1β neutralization. *Top panels*: *green*, P2X7R-EGFP, *red*, isolectin GS-IB_4_, *blue*, DAPI nuclear stain. *Bottom panels*: green, P2X7R-EGFP, blue, DAPI nuclear stain. *Scale bars*, 20 μm. **c** Primary hippocampal cultures expressing P2X7R-EGFP with caspase 1 inhibited (caspase 1−) (*n* = 40) showed a decrease in total number of cells (DAPI positive) compared to the P2X7R-EGFP-expressing control (caspase 1+) (*n* = 40) cultures with no caspase 1 inhibition. **d** The number of transfected cells expressing P2X7R-EGFP was smaller in cultures where caspase 1 was inhibited (caspase 1–) (*n* = 40) compared to the controls (caspase 1+) (*n* = 40) with no inhibition of caspase 1. **e** The absolute number of microglia per imaging field was decreased in the P2X7R-EGFP-expressing cultures with caspase 1 inhibited (caspase 1−) (*n* = 40) compared to the controls (caspase 1+) (*n* = 40). **f** Primary hippocampal cultures expressing P2X7R-EGFP with IL-1β activity neutralized (IL-1β−) with the aid of a neutralizing antibody (*n* = 40) showed a decrease in the total number of cells (DAPI positive) compared to the P2X7R-EGFP-expressing control (IL-1β+) (*n* = 40) cultures with no IL-1β neutralization. **g** As a proportion of the total microglial pool (identified by isolectin GS-IB_4_ and DAPI nuclear counter-staining) less activated microglia were evident in cultures expressing P2X7R-EGFP (*n* = 40) with the activity of IL-1β neutralized (IL-1β–) compared to the controls with no neutralization (IL-1β+) (*n* = 40). **h** The absolute number of microglia per imaging field was decreased in the P2X7R-EGFP-expressing cultures with IL-1β neutralization (IL-1β−) (*n* = 40) compared to the controls (IL-1β+) (*n* = 40). Imaging field dimension, 230 × 230 μm. *p < 0.05, Student’s *t* test

Processing of IL-1β to its active form was inhibited by blocking the intracellular actions of caspase 1 with a specific antagonist (N-Acetyl-TRP-GLU-His-Asp-AL). As a result, the total number of cells (Fig. 7c), the number of activated microglia expressing P2X7R (Fig. 7d), and the number of microglia (Fig. 7e) were all reduced. These results highlight the production of the active IL-1B to be an important step in P2X7R-mediated activation of microglia.

### Microglial activation and proliferation is reduced by neutralization of extracellular IL-1β

When the potential effects of the extracellular, active form of IL-1β were abolished, microglial activation and proliferation were markedly reduced. Addition of an IL-1β neutralizing antibody to cultures expressing exogenous wild-type P2X7R caused significant reduction in the total number of cells (Fig. 7f*)*, the number of activated microglia expressing exogenous P2X7R (Fig. 7g), and the number of microglia (Fig. 7h).

## Discussion

Here, we reveal that microglial vesicles adjacent to the plasma membrane co-express P2X7R and IL-1β and are likely to be of the lysosomal family of vesicles. Lysosomal vesicular exocytosis was more pronounced in activated microglia expressing wild-type P2X7R as opposed to those expressing a non-pore-forming mutant (*P2X7G345Y*). Cultures expressing wild-type P2X7R contained higher quantities of the extracellular (released) form of IL-1β than those expressing the non-pore-forming mutant. We also reveal that IL-1β is a key cytokine mediating the trophic effects of the P2X7R pore in microglia, as inhibition or neutralization of IL-1β resulted in significant reduction in P2X7R pore-driven microglia activation and proliferation. These findings highlight an important role for P2X7R pore formation and consequent production and release of IL-1β, indicating the critical role that is played by P2X7R pore formation in the hierarchy of neuroinflammation.

### Activated microglia contain sub-plasma membrane vesicles that express IL-1β and P2X7R and these vesicles are of lysosomal origin

In this study, we showed that activated microglia displayed “nodular structures” as outpouchings of the cell cytoplasm containing discrete vesicles ranging 100–200 μm in diameter. These vesicles expressed P2X7R, IL-1β, and the lysosomal maker LAMP-1.

The proposed mechanisms of release of IL-1β include (i) exocytosis of secretory lysosomes, (ii) release of membrane spanning microvesicles derived from plasma membrane blebs, (iii) release of membrane-delimited exosomes derived from multivesicular bodies formed by invagination of endosomes, (iv) exocytosis of autophagosomes, and (v) direct release to the extracellular environment subsequent to cell death [15]. Our data support the lysosomal mode of release. Lysosomes were previously considered as end organelles in protein degradation, but recent studies have shown that lysosomes may also play roles in cellular signal transduction and secretion of signaling molecules [35, 36]. In cells of hemopoietic origin such as microglia, the lysosome can act as a secretory organelle [35, 36]. Previously it has been shown that FM143 and AM143 label lysosomal vesicles (by co-localizing with lysosomal markers LAMP1 and cathepsin D), but not endosomes (no evidence of co-localization with endosomal marker EAA1) [30]. Our data, showing lysosomal localization of IL-1β and co-expression with P2X7R, indicates that it is likely that lysosomal exocytosis is involved in IL-1β release from microglia.

Expression of vesicles containing bioactive substances and their calcium-induced exocytosis were long thought to be a property exclusive to neurons and neuroendocrine cells. However, significant evidence in the last 10 years indicates exocytosis of various factors from glia, in a process now called gliotransmission [37]. In astrocytes, FM1-43 only labels lysosomes, calcium-dependent exocytosis of lysosomes has been revealed by confocal microscopy [29, 38], and it was proposed that the lysosome is the major type of vesicle for calcium-regulated exocytosis. Thus, we reveal that microglia are similarly competent in undergoing exocytosis, and the process is P2X7R pore dependent.

### The release of IL-1β is P2X7R pore dependent

In agreement with our findings, there are numerous prior reports that the release of IL-1β is dependent on P2X7R function [2, 5, 8, 10, 16, 39]. Grahames and colleagues reported that exogenous application of ATP resulted in the release of IL-1β from human monocytes, and the pharmacological profile was consistent with that of P2X7R stimulation [7]. Similarly, macrophages derived from P2X7R knockout mice did not release IL-1β in response to ATP stimulation [8]. Moreover, oxATP, an irreversible antagonist of both ion channel and pore conductances of P2X7R [34] was found to abolish ATP-induced IL-1β release from human macrophages [4]. Mariathasan and colleagues described a mechanism whereby P2X7R acted up-stream of the inflammasome to ultimately lead to the production and release of IL-1β [40].

In addition, P2X7R stimulation was also linked to the activation of NF-kB [41], a factor whose activity is thought to lead to enhanced transcription of IL-1β gene. As the experimental conditions used in all of these studies would involve stimulation, or conversely, antagonism of both ion channel and pore conductance, it is not possible to distinguish whether P2X7R channel or pore conductance was predominantly responsible for the production and release of IL-1β. Although recognized for its pro-death properties [5, 42–52], the contribution of the P2X7R pore to trophic cellular effects was largely unknown. Recently, we revealed that up-regulation of the P2X7R pore lead to microglial activation and proliferation [20] and now reveal IL-1β to be a key extracellular mediator of the trophic effects. Interestingly, Di Virgilio and colleagues reported that amyloidβ peptide (Aβ_25–35_) triggered increases in intracellular Ca^2+^, IL-1β release, and plasma membrane permeabilization in microglia derived from wild-type but not P2X7R-deficient mice [2]. However, the results of this study, although consistent with activation of pore conductance, did not distinguish the specific contributions of the P2X7R pore and channel.

An attempt at this distinction between P2X7R channel and pore activities was made with P2X7R truncation mutants, where segments of the C terminus are removed to produce a mutant receptor without the capacity to form pores [53]. However, we have previously shown with P2X7R truncation mutants that while the C terminus is critical for P2X7R pore activity (the receptor needing 95 % of its C terminus to retain its pore function), the majority of P2X7R C-terminal truncation mutants suffer from altered folding, trafficking, and many rather than reaching their final plasmalemmal destination, aggregate in intracellular organelles [52]. In addition, the C-terminal domain of P2X7R is imperative in the interaction of the receptor with intracellular signaling cascades [54]. By deleting these regions, one would inevitably abolish the ability of the receptor to participate in potentially important cellular signaling cascades. In the current study, we used instead P2X7R*G345Y*, a full-length, point mutant of P2X7R, with normal trafficking and expression properties with intact channel function but ablated pore-forming capacity [20] to show that the release of IL-1β was P2X7R pore dependent.

Our data indicates that the release of the cytokine is likely via lysosomal exocytosis, rather than cell lysis as suggested for macrophages [55], as P2X7R pore activity enhances microglial proliferation with no evidence of cytolysis [20]. In agreement, others have unequivocally dissociated the P2X7R-induced release of IL-1β from cytolysis [56] in human monocytes [57], human macrophages [4], and mouse peritoneal macrophages [3].

Li and colleagues found that pore dilation results from time-dependent alterations in the concentration of intracellular ions [58]. The P2X7R pore is unlikely to function as a transporter or conduit for the cytokine, due to the difference in size of the pore (≤900 Da) and the immature (34 kDa) and mature (17 kDa) forms of IL-1β [59]. P2X7R pore formation may instead lead to activation of intracellular signaling cascades such as MAPK or Rho-effector kinase, or induction of NF-kB, ultimately leading to transcription, as well as caspase 1-induced processing and release of IL-1β via lysosomal exocytosis.

### The trophic effects of P2X7R pore are mediated by IL-1β

Our findings are consistent with previous reports that basal proliferation of microglia is stimulated by IL-1β or tumor necrosis factor-α (TNF-α) [60]. The literature linking microglial activation and excess IL-1β release, with the pathogenesis of a number of neuroinflammatory conditions is extensive [61, 62]. Of particular note are observations that human IL-1 is elevated in cerebrospinal fluid derived from patients with brain injury [63], and the increased levels reported within brain lesions from patients with Alzheimer’s disease, multiple sclerosis, and HIV-associated dementia [64–67], and in CSF in Parkinson’s disease and Creutzfeldt-Jakob disease [68, 69].

Once released, IL-1β in turn can exert autocrine actions on microglia, and paracrine actions on astrocytes [70]. IL-1β can cause the production of a number of other proinflammatory bioactive substances (nitric oxide and IL-6) from astrocytes and microglia in culture [71, 72]. In turn, these substances have been implicated in the propagation of a number of CNS injuries and diseases [73].

## Conclusions

In summary, the studies in this research showed that the release of IL-1β, while dependent on P2X7R function, requires P2X7R pore activity and is independent of cytolysis. Our results support a model where up-regulation of P2X7R and stimulation of pore activity leads to microglial activation and subsequent release of IL-1β through lysosomal exocytosis. This cytokine can potentially maintain or accelerate the trophic responses in microglia, with consequent deleterious cycles of neuroinflammation and neurodegeneration. There is evidence that polymorphisms in P2X7R that are associated with a loss or reduction of P2X7R function are associated with a decreased sensitivity to inflammation [9, 74]. Given the highly documented role of IL-1β in many neuropathological scenarios, our results suggest that specific manipulation of P2X7R pore could be utilized to reduce the degree of microglial activation, and this therapeutic strategy holds promise in the setting of Alzheimer’s and other neurodegenerative diseases, as well as neoplastic conditions where effective interventions are currently lacking.

## Abbreviations

BzATP, 2′3′-O-(4-benzoylbenzoyl)-ATP (agonist of P2X7R); FM1-43, fixable fluorescent probe used to study vesicular recycling; IL-1β, interleukin-1beta (cytokine); P2X7R, P2X7 receptor; P2X7R*G345Y*, P2X7R with glycine to thyrosine point mutation at position 345

## Additional file

Additional file 1: Prior to the commencement of the video, activated microglia expressing P2X7R-EGFP were loaded with FM1-43. When the cells were then stimulated with BzATP (100μM) (at the commencement of the video), vesicles of ~200μm diameter appeared on the surface of the cell with subsequent ‘release events’ (seen in the video as fusion of green ‘beaded’ structures to the surface membrane). Exocytosis was relatively fast with application of the agonist, reaching its maximum by mid-section of the video. The entire duration (real-time) lapsed during the video is 24.75 minutes. In total 100 images (1 image every 15 seconds) were captured for this experiment, and played at approximately 10 images per second. (MOV 6.25 mb)
